# Bioinspired Catechol-Based Systems: Chemistry and Applications

**DOI:** 10.3390/biomimetics2040025

**Published:** 2017-12-20

**Authors:** Marco d’Ischia, Daniel Ruiz-Molina

**Affiliations:** 1 Department of Chemical Sciences, University of Naples Federico II, Via Cintia 4, I-80126 Naples, Italy; 2 Catalan Institute of Nanoscience and Nanotechnology (ICN2), CSIC and BIST, Campus UAB, Bellaterra, 08193 Barcelona, Spain

Catechols are widely found in nature taking part in a variety of biological functions, ranging from the aqueous adhesion of marine organisms to the storage of transition metal ions. This has been achieved thanks to their (i) rich redox chemistry and ability to cross-link through complex and irreversible oxidation mechanisms, (ii) excellent chelating properties, and (iii) the diverse modes of interaction of the vicinal hydroxyl groups with all kinds of surfaces of remarkably different chemical and physical nature [1]. Therefore, guided by such interest, catechol-based systems have been subject in recent years to intense research (at the laboratory scale) aimed at mimicking and translating these natural concepts into new functional adhesives and coatings with enhanced properties.

This Special Issue collects contributions from different laboratories working on both basic research and applications of bioinspired catechol systems presented by cutting edge specialists in this growing field. Taking advantage of its open access publication, this collection of papers, influenced by biomimetic approaches, will bring about new avenues for new research and innovative solutions in biomedicine and technology. Main topics addressed in the field of basic catechol chemistry include (i) a computational investigation by Barone et al. [2] of noncovalent interactions in catechol dimers, which are of central importance in determining the overall properties of catechol base systems, (ii) a theoretical analysis of indole-based porphyrin structures proposed as a model for eumelanin biopolymers (Crescenzi et al. [3]), and (iii) a detailed insight into the mechanism of the antioxidant activity of quercetin in water by Amorati et al. [4]. Of both basic and applicative interest for adhesion is the study by Hamada et al. [5] addressing the issue of whether mussels manage byssus mechanical properties via control of catechol chemistry. The design of films for surface functionalization and energy applications based on polydopamine-inorganic and polydopamine-organic composites is reviewed by Ball [6]. The potential of a cross-linking reaction between catechol and hexamethylenediamine for surface functionalization and coating under oxidative conditions is demonstrated by Suarez-Garcia et al. [7]. Catechol-containing hydrogels with enhanced gluing properties for tissue engineering are reported by Feng et al. [8]. Sousa and Mano [9] synthesized cell-adhesive membranes for bone tissue engineering via a mussel-inspired conjugated polymer obtained by covalent modification of hyaluronic acid with dopamine. Amin et al. [10] studied melanin-mimetic nanoparticles based on polydopamine for multimodal cell imaging, opening interesting perspectives for drug delivery applications and surface chemistry-dependent cellular interactions. The scope of catechol–chitosan redox-capacitors and other systems for chemical information and signal processing is illustrated by Kim et al. [11]. In the field of bioinspired bioactive compounds, Micillo et al. [12] designed a lipoyl–caffeic acid conjugate as a new type of tyrosinase inhibitor for the control of melanogenesis. Ramazzotti et al. [13] report the anti-aggregating properties of five biomimetic 4-thiaflavanes on an amyloid model, suggesting further studies of this class of compounds as anti-amyloid agents.

Integrating more than replacing the many excellent reviews, the present collection will provide the reader with a concise panorama of the status quo and perspectives in the increasingly expanding field of basic and applied research on bioinspired catechol systems. It is clear that the interest for catechol-based materials is experiencing a steady burst, perfectly represented by polydopamine (two contributions in this special issue deal with this research area). Several patents based on bioinspired catechol systems and different products are already commercialized and available the market. We believe that this special issue may fulfill an important function in promoting biomimetic catechol chemistry for an increasing range of applications.
